# Genetically proxied gut microbiota, gut metabolites with risk of epilepsy and the subtypes: A bi-directional Mendelian randomization study

**DOI:** 10.3389/fnmol.2022.994270

**Published:** 2022-11-03

**Authors:** Yuzhen Ouyang, Yu Chen, Ge Wang, Yanmin Song, Haiting Zhao, Bo Xiao, Zhuanyi Yang, Lili Long

**Affiliations:** ^1^Department of Neurology, Xiangya Hospital, Central South University, Changsha, China; ^2^National Clinical Research Center for Geriatric Disorders, Xiangya Hospital, Central South University, Changsha, China; ^3^Clinical Research Center for Epileptic Disease of Hunan Province, Central South University, Changsha, China; ^4^Department of Emergency, Xiangya Hospital, Central South University, Changsha, China; ^5^Department of Neurosurgery, Xiangya Hospital, Central South University, Changsha, China

**Keywords:** gut microbiota, gut metabolites, epilepsy, bi-directional Mendelian randomization study, causality

## Abstract

**Background:**

An increasing number of observational studies have revealed an association among the gut microbiota, gut metabolites, and epilepsy. However, this association is easily influenced by confounders such as diet, and the causality of this association remains obscure.

**Methods:**

Aiming to explore the causal relationship and ascertain specific gut microbe taxa for epilepsy, we conducted a bi-directional Mendelian randomization (MR) study based on the genome-wide association study (GWAS) data of epilepsy from the International League Against Epilepsy, with the gut microbiota GWAS results from MiBioGen, and summary-level GWAS data of gut microbiota-dependent metabolites trimethylamine N-oxide and its predecessors.

**Results:**

Nine phyla, 15 classes, 19 orders, 30 families, and 96 genera were analyzed. A suggestive association of host-genetic-driven increase in family Veillonellaceae with a higher risk of childhood absence epilepsy (odds ratio [OR]: 1.033, confidential interval [CI]: 1.015–1.051, *P*_*IVW*_ = 0.0003), class Melainabacteria with a lower risk of generalized epilepsy with tonic-clonic seizures (OR = 0.986, CI = 0.979–0.994, *P*_*IVW*_ = 0.0002), class Betaproteobacteria (OR = 0.958, CI = 0.937–0.979, *P*_*IVW*_ = 0.0001), and order Burkholderiales (OR = 0.960, CI = 0.937–0.984, *P*_*IVW*_ = 0.0010) with a lower risk of juvenile myoclonic epilepsy were identified after multiple-testing correction. Our sensitivity analysis revealed no evidence of pleiotropy, reverse causality, weak instrument bias, or heterogeneity.

**Conclusion:**

This is the first MR analysis to explore the potential causal relationship among the gut microbiota, metabolites, and epilepsy. Four gut microbiota features (two class levels, one order level, and one family level) were identified as potential interventional targets for patients with childhood absence epilepsy, generalized epilepsy with tonic-clonic seizures, and juvenile myoclonic epilepsy. Previous associations in numerous observational studies may had been interfered by confounders. More rigorous studies were needed to ascertain the relationship among the gut microbiota, metabolites, and epilepsy.

## Introduction

Epilepsy is a common neurological disease characterized by recurrent unprovoked seizures, besetting over 50 million patients worldwide, with an incidence rate of 50–70 per 100,000 people (Deuschl et al., 2020). With the main manifestation of epileptic seizures and abnormal electroencephalogram results, epilepsy is a disease with strong heterogeneity, which can be further classified into several subtypes such as focal epilepsy and generalized epilepsy. The etiology of epilepsy is also complex as it involves genetic, structural, infectious, metabolic, immune, and unknown factors (Beghi, 2020).

Recently, the mutual interaction between the gut microbiota and the human body is revealed, and the gut microbiota is considered as a promising therapeutic target through probiotic supplements and fecal microbiota transplantation (FMT) (Gomaa, 2020). Among these microbiome-to-host interactions, the central nervous system (CNS) and gut microbiota communication, termed the microbiota–gut–brain (MGB) axis, is a research hotspot, and its interaction routes are related to metabolites, immune responses, and the enteric nervous system (Cryan et al., 2019). The potential functions of the MGB axis in psychiatric and CNS disorders have been previously summarized (Iannone et al., 2019; Socała et al., 2021). Epilepsy, one of the most common diseases of the CNS, is also related to the gut microbiota and metabolites through the MGB axis at both laboratory and clinical levels (Holmes et al., 2020; Ding et al., 2021). For example, a distinct gut microbiome profile has been detected in patients with epilepsy, especially in those with anti-seizure medication resistance (Peng et al., 2018; Şafak et al., 2020). Furthermore, high-fat ketogenic diet (KD) is recommended for patients with epilepsy, diet-related gut microbiome alterations are observed with an anti-seizure effect (Olson et al., 2018; Ang et al., 2020). Therefore, the gut microbiota is a promising biomarker and therapeutic target for epilepsy (De Caro et al., 2019; Arulsamy and Shaikh, 2022; Russo, 2022). However, the causality among the gut microbiota, metabolites, and epilepsy remains unclear and requires more direct evidence.

Randomized controlled trials (RCTs) are the gold standard for exploring causal relationships. However, an RCT is not only costly but also difficult for investigating the gut microbiome and neurological disorders because of potential confounders. Meanwhile, the Mendelian randomization (MR) study is an alternative tool for exploring the causal relationship between exposure and outcome, utilizing single nucleotide polymorphisms (SNPs) as instrumental variables (IVs) (Smith and Ebrahim, 2003). An MR study has several types such as two-sample MR, two-step MR, and bi-directional MR. The two-sample MR refers to MR based on the exposure and outcome using a genome-wide association study (GWAS) dataset without overlap. The bi-directional MR is utilized to test the effect of exposure on the outcome, and the outcome on exposure by retrieving different IVs from exposure or outcome datasets to ascertain the robustness of direction. With increasing publicly available GWAS data, an MR study is more feasible for conducting epidemiological research. Moreover, abundant GWAS data in the gut microbiome, gut-microbiome metabolites, and epilepsy have been reported recently, thus providing the research foundation of our MR analysis.

In this study, we conducted the first bi-directional MR analysis to examine the causal relationship among the gut microbiome, metabolites, and epilepsy following the “STROBE-MR” guidelines (Skrivankova et al., 2021a,b). The summary statistics of epilepsy, gut microbiota, and metabolites are derived from the International League Against Epilepsy (ILAE) consortium and large-scale GWAS data. This research not only improves our understanding of the mutual interaction among the gut microbiota, metabolites, and epilepsy but also reveals the research direction.

## Methods

### Study design and data sources

**FIGURE 1:**
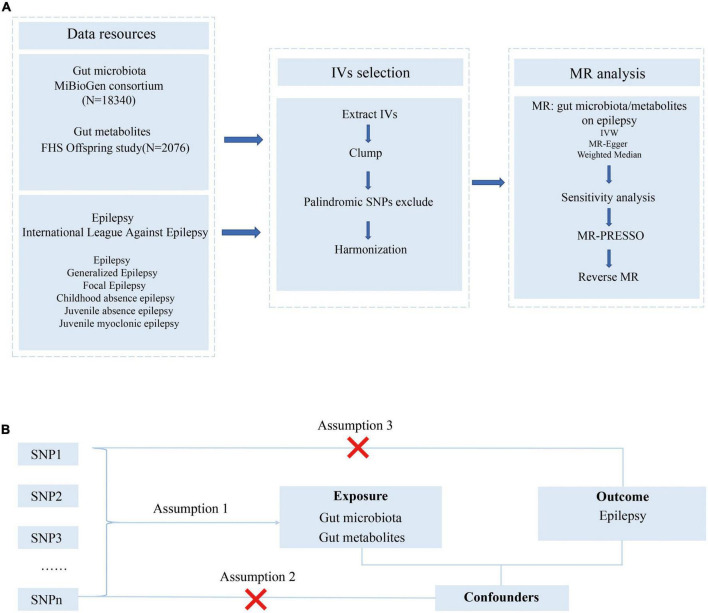
Study design and Mendelian randomization core assumption. **(A)** Data resource and study design of our bi-directional MR. **(B)** Three assumptions in the Mendelian randomization study.

The study flowchart is presented in Figure 1A. We conducted a bidirectional MR study to investigate the causal relationship among the gut microbiota, metabolites, and epilepsy. First, genetic variants from previous GWAS summary-level data are retrieved and used as IVs. Then, a two-sample MR is conducted using the R software (4.1.3) following the guideline of the R package ‘‘two-sample MR’’ (0.5.6)^1^ including three MR methods. Several sensitivity analyses, such as the pleiotropy test, heterogeneity test, and leave-one-out analysis, are performed sequentially. Finally, we adopted a reverse MR method to explore whether a bidirectional relationship exists among epilepsy, the gut microbiota, and metabolites.

Genome-wide association study (GWAS) statistics summary-level data of the gut microbiome have been generated from the largest genome-wide meta-analysis to date, the MiBioGen study (Kurilshikov et al., 2021). This MiBioGen consortium curated and analyzed 16S faucal microbiome data and genome-wide genotypes from 24 cohorts (18,340 individuals) and identified 31 loci affecting the gut microbiome with genome-wide significance (*P* < 5 × 10^–8^). As for gut metabolite data, we leveraged GWAS summary statistics of the human metabolome in a community-based cohort containing 2076 participants (Rhee et al., 2013). GWAS summary statistics for epilepsy are retrieved from the OpenGWAS database API^2^ (Elsworth et al., 2020). The GWAS data in this database included epilepsy (GWAS ID: ieu-b-8), genetic generalized epilepsy (ieu-b-9), focal epilepsy (ieu-b-10), focal epilepsy-documented lesion negative (ieu-b-11), juvenile absence epilepsy (ieu-b-12), childhood absence epilepsy (ieu-b-13), focal epilepsy-documented hippocampal sclerosis (ieu-b-14), focal epilepsy-documented lesions other than hippocampal sclerosis (ieu-b-15), generalized epilepsy with tonic-clonic seizures (ieu-b-16), and juvenile myoclonic epilepsy (ieu-b-17) based on the ILAE classification position paper on epilepsies (Scheffer et al., 2017; Ilae Consortium, 2018). Detailed information on the dataset used, without overlap between the exposure and outcome data, is summarized in Table 1. Additional information, including demographic characteristics, eligibility criteria, and ethics approval can be found in the original article (Ilae Consortium, 2018).

**TABLE 1:** Characteristics of included GWAS summary-level data of epilepsy, gut microbiota, and gut metabolites.

Trait	Consortium of study	Sample size	Population	Journal	Year
Epilepsy	ILAE				
Epilepsy (All documented cases)	ieu-b-8	44889 (case: 15212, control: 29677)	Mixed	Nat Com.	2018
Generalized epilepsy (All documented cases)	ieu-b-9	33446 (case: 3769, control: 29677)			
Focal epilepsy (All documented cases)	ieu-b-10	39348 (case: 9671, control: 29677)			
Focal epilepsy-documented lesion negative	ieu-b-11	32393 (case: 2716, control: 29677)			
Juvenile absence epilepsy	ieu-b-12	30092 (case: 415, control: 29677)			
Childhood absence epilepsy	ieu-b-13	30470 (case: 793, control: 29677)			
Focal epilepsy-documented hippocampal sclerosis	ieu-b-14	30480 (case: 803, control: 29677)			
Focal epilepsy-documented	ieu-b-15	32747 (case: 3070, control: 29677)			
lesion other than hippocampal sclerosis					
Generalized epilepsy with tonic-clonic seizures	ieu-b-16	29905 (case: 228, control: 29677)			
Juvenile myoclonic epilepsy	ieu-b-17	30858 (case: 1181, control: 29677)			
Gut microbiota	MiBioGen	18340	Mixed	Nat Genet.	2021
Gut metabolites	FHS	2076	European	Cell Metab.	2013

GWAS, Genome-Wide Association Study; ILAE, International League Against Epilepsy; FHS, Framingham heart study.

### Instrumental variables selection

Bacterial taxa were classified and analyzed at six levels (phylum, class, order, family, genus, and species) based on SNPs available in the gut microbiome GWAS summary data. Candidate IVs were identified at a significance level of *P* < 1.0 × 10^–5^ according to the previously published studies (Sanna et al., 2019; Ni et al., 2021). The parameters of the clump function in the R package were set at *r*^2^< 0.1 and kb = 500 kb, guaranteeing the independence of each IV, which was the same as in a previous publication, to minimize the impact of linkage disequilibrium violating the randomized allele allocation (Ni et al., 2021). Furthermore, as our significance level was set at *P* < 1.0 × 10^–5^, the F statistic was used to exclude the weak instrument bias violating the first assumption of MR (Burgess and Thompson, 2011). Lastly, the palindromic SNPs were also excluded from the MR. The IVs adopted in this study are listed in Supplementary Table 1. Similarly, IVs of gut metabolites were extracted under a suggestive significance level of *P* < 5.0 × 10^–5^ (Zhuang et al., 2021). The parameters of the clump function in the R package were set at *r*^2^< 0.2 and kb = 10,000 kb (Zhuang et al., 2021).

For the outcome data, we collected epilepsy GWAS data from the MR database (see text footnote 1). The gut microbiome, metabolites, and epilepsy data were harmonized for subsequent MR. As the exposure and outcome GWAS datasets were large-scale GWAS research, the threshold of minor allele frequency was set at 0.01.

### Mendelian randomization study

As presented in Figure 1B, this MR was conducted following the MR model with selected IVs in the previous step, conforming to three assumptions as follows: (1) SNPs were robustly associated with the gut microbiome/metabolites; (2) SNPs were not associated with confounders; (3) SNPs do not affect the epilepsy outcomes except through the potential effects of the gut microbiome or metabolites.

A two-sample MR analysis was conducted using three primary methods: inverse variance weighted median (IVW), weighted median, and MR Egger to evaluate the causal relationship among the gut microbiome, metabolites, and epilepsy (Bowden et al., 2015, 2016; Burgess et al., 2015). The MR analysis was conducted using two-sample MR packages according to the developers’ guidelines. IVW was a classic method based on the meta-analysis of each SNPs Wald ratio, while the weighted median mode calculated the median effects of SNPs. The MR Egger analysis, albeit with lower statistical power than IVW, can be applied in the presence of horizontal pleiotropy. Additionally, we conducted an MR-PRESSO test using the MR-PRESSO R package (1.0) to evaluate whether a horizontal pleiotropy effect violates the assumption of MR (Verbanck et al., 2018). Based on the MR-PRESSO Global test for overall horizontal pleiotropy and outlier test for each SNP pleiotropy significance evaluation, outlier SNPs were removed until the *P*-value of the global test remained >0.05. Moreover, the multiple-testing significance threshold at each level (phylum, class, order, family, and genus) was set as 0.05/n, where n was the effective number of independent bacterial taxa at each taxonomic level. However, owing to the sample size and restricted power of the gut microbiota GWAS data, IVs at the species level were insufficient for MR analysis. Therefore, we conducted MR at the phylum, class, order, family, and genus levels.

### Sensitivity analysis

The MR-Egger regression and MR-PRESSO tests were conducted to exclude potential pleiotropy. The Q test in the IVW test was performed to evaluate the heterogeneity of results. The leave-one-out analysis excluded SNPs individually and recomputed the effect to test the robustness of the results. The MR Steiger directionality test was adopted to explore the robustness of the causality direction.

### Reverse Mendelian randomization analysis

A reverse MR analysis was conducted to explore the reverse causality from epilepsy (as exposures) to gut microbiota and metabolites (as outcomes). The procedure was the same as the abovementioned protocol for the two-sample MR.

This bidirectional MR and sensitivity analysis adhered to the guidelines of the two-sample MR and MR-PRESSO packages.

## Results

### Two-sample Mendelian randomization of gut microbiota (exposure) on epilepsy (outcome)

Twelve exposure and outcome datasets were acquired for MR analysis with detailed information such as the consortium, sample size, and population (Table 1). Under a suggestive significance level of *P* < 1 × 10^–5^, the significant SNPs were selected from the GWAS summary data of gut microbiota in the nine phyla, 16 classes, 20 orders, 35 families, and 96 genera. After clumping and harmonization, an MR analysis was conducted between each pair of exposure and outcome to explore causality. The significant threshold for each level was corrected based on multiple testing as follows: phylum *P* = 5.56 × 10^–3^ (0.05/9); class *P* = 3.13 × 10^–3^ (0.05/16); order *P* = 2.50 × 10^–3^ (0.05/20); family *P* = 1.43 × 10^–3^ (0.05/35); genus *P* = 5.21 × 10^–4^ (0.05/96); and gut metabolites *P* = 1.25 × 10^–2^ (0.05/4).

**FIGURE 2:**
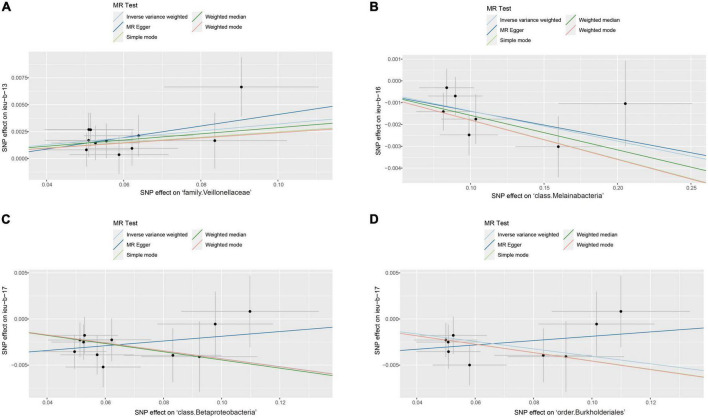
Scatter plots of significant causality of the gut microbiota and epilepsy. **(A)** Scatter plots of the family Veillonellaceae on childhood absence epilepsy. **(B)** Scatter plots of the class Melainabacteria on generalized epilepsy with tonic-clonic seizures. **(C)** Scatter plots of the class Betaproteobacteria and **(D)** The order Burkholderiales on juvenile myoclonic epilepsy. The lines move obliquely upward from left to right exhibiting a positive correlation between the gut microbiota and epilepsy with horizontal and vertical lines indicating the 95% confidence interval of each association. The lines with a negative correlation are inclined downward from left to right, indicating a protective effect of the gut microbiota on epilepsy.

**FIGURE 3:**
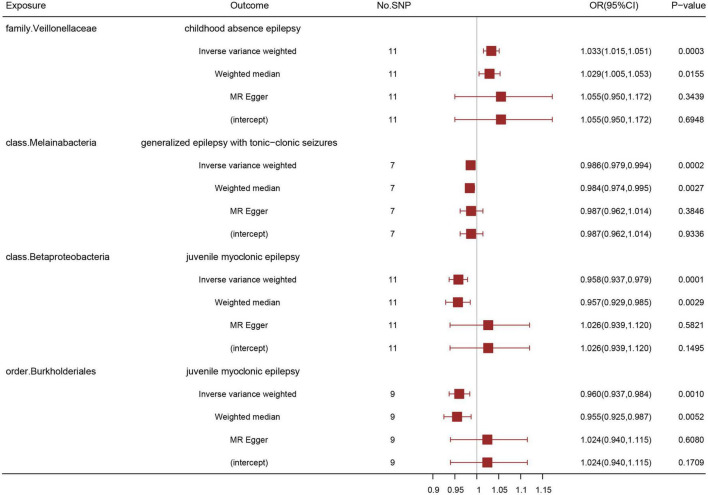
Association of genetically predicted gut microbiota with epilepsy by three different MR methods: inverse variance weighted, weighted median, and MR Egger. A positive correlation between the family Veillonellaceae and childhood absence epilepsy, negative correlations between the class Melainabacteria and generalized epilepsy with tonic-clonic seizures, between the class Betaproteobacteria and juvenile myoclonic epilepsy, and between the order Burkholderials and juvenile myoclonic epilepsy were suggested. OR: odds ratio; CI: confidential interval.

After data preprocessing, an MR analysis was performed for each pair of exposure (gut microbiota) and outcome (epilepsy) based on three MR methods (IVW, weighted median, and MR Egger). Under the corrected significant threshold, four correlations between gut microbiome features and epilepsy were identified using the IVW method (Table 2) as follows: family Veillonellaceae with a higher risk of childhood absence epilepsy (*P*_IVW_ = 3.00 × 10^–4^), class Melainabacteria with a lower risk of generalized epilepsy with tonic-clonic seizures (*P*_IVW_ = 2.00 × 10^–4^), and class Betaproteobacteria (*P*_IVW_ = 1.18 × 10^–4^) and order Burkholderiales (*P*_IVW_ = 1.03 × 10^–3^) with a lower risk of juvenile myoclonic epilepsy. These correlations are depicted in scatter and forest plots in Figures 2, 3, respectively. More information on single SNP is summarized in Supplementary Table 1.

**TABLE 2:** Gut microbiota on epilepsy result.

Exposure	Outcome (ID)	No. SNP	R2	F	IVW		WME		MR-Egger		MR-PRESSO	
								
					B	P	B	P	b	P	B	P
Family Veillonellaceae	ieu-b-13	11	1.58%	20.73	0.0321	0.0003	0.0287	0.0155	0.0535	0.3439	0.0304	0.0003
Class Melainabacteria	ieu-b-16	7	3.53%	18.04	–0.0138	0.0002	–0.0158	0.0027	–0.0127	0.3846	–0.0100	0.0243
Class Betaproteobacteria	ieu-b-17	11	1.44%	20.10	–0.0429	0.0001	–0.0444	0.0029	0.0256	0.5821	–0.0429	0.0005
Order Burkholderiales	ieu-b-17	9	1.20%	20.29	–0.0405	0.0010	–0.0455	0.0052	0.0235	0.6080	–0.0405	0.0034

In the heterogeneity (IVW test and MR-Egger regression), pleiotropy (MR-PRESSO test and MR-Egger regression test) and weak instrument bias tests (F statistic), no evidence of heterogeneity, pleiotropy, or weak instrument bias was noted. Additional details are summarized in Supplementary Table 2. Furthermore, the MR Steiger directionality test revealed a robust direction from the gut microbiota to epilepsy in all results. The leave-one-out sensitivity analysis illustrated the robustness of our results, as no single SNP drives causal association (Supplementary Figure 1). Funnel plots of these four significant results excluded a potential bias (Supplementary Figure 2).

### Reverse Mendelian randomization analysis of epilepsy (exposure) on gut microbiota (outcome)

With a significant *P*-value set at 1 × 10^–5^, IVs were extracted from significant epilepsy GWAS datasets in previous MR analysis of gut microbiota on epilepsy; however, no significant results were identified in this reverse MR analysis. The reverse analysis indicated the absence of causality from epilepsy to the gut microbiota, which was in accordance with our MR steiger results.

### Bi-directional Mendelian randomization analysis Mendelian randomization of gut metabolites and neurological disorders

In the gut metabolite and epilepsy MR analysis, the SNPs concerning carnitine, choline, trimethylamine N-oxide (TMAO), and betaine were extracted from the GWAS summary data. With a genome-wide significance level set at *P* < 5 × 10^–5^, which was the same as the parameters in previous publications, SNPs were extracted for subsequent MR analysis after clumping (*r*^2^ = 0.2, kb = 10,000) and harmonization (Zhuang et al., 2021). Similar to the gut microbiota and epilepsy MR analysis, three MR methods (IVW, weighted median, and MR-Egger) were adopted to explore the potential causality with multiple sensitivity analyses. No significant causality between gut metabolites and epilepsy was found. For reverse MR analysis, IVs extracted from epilepsy under significance levels were not identified in the gut metabolite GWAS dataset.

All results on phylum, class, order, family, genus, or metabolite levels are summarized in Supplementary Tables 3–8.

## Discussion

To the best of our knowledge, this is the first MR study to reveal the potential causal relationship among the gut microbiome, metabolites, and epilepsy based on large GWAS summary-level data. The potential contributory or protective effect of the family Veillonellaceae on childhood absence epilepsy, class Melainabacteria on generalized epilepsy with tonic-clonic seizures, class Betaproteobacteria, and order Burkholderials on juvenile myoclonic epilepsy are demonstrated in this MR analysis.

The association between the gut microbiota and epilepsy has been summarized in a systematic review published in 2020 with an increasing number of studies published over the past two years; however, the gut microbiota alteration is inconsistent in all the studies (Arulsamy et al., 2020). Some studies have revealed that the richness of numerous gut microbiomes, such as Proteobacteria and Fusobacterial phyla, as possible biomarkers for epilepsy diagnosis increased in patients with epilepsy (Şafak et al., 2020; Dong et al., 2022). Regarding the gut microbiota characteristics of patients with drug-resistant epilepsy (DRE), the Firmicutes phylum is commonly increased in DRE, and bifidobacteria exhibits a potential protective function against epilepsy (Peng et al., 2018; Lee et al., 2020, 2021). In addition to the altered gut microbiota composition of patients with epilepsy, KD therapy, commonly recommended for patients with DRE, also exerts a therapeutic effect due to its influence on the gut microbiota (Fan et al., 2019; Tang et al., 2021). Decreased Proteobacteria and Firmicutes and increased Bacteroidetes are commonly observed after KD therapy (Xie et al., 2017; Zhang et al., 2018; Gong et al., 2021). In addition to KD therapy, FMT is also a promising strategy for epilepsy, and a case report has demonstrated that epilepsy in a girl with Crohn’s disease was cured after FMT (He et al., 2017). Therefore, we summarized the diverse gut microbiota changes to directly exhibit these observational results of the gut microbiota and epilepsy in Supplementary Table 9.

The abovementioned studies have confirmed the close relationship between the gut microbiota and epilepsy, and KD therapy may alter the bacteria to further control seizures. However, a direct causal relationship is unclear and may be affected by confounding factors. Intriguingly, numerous correlations summarized in Supplementary Table 9 are not identified in our MR analysis after multiple-testing corrections for several reasons. Supplementary Tables 3–8 exhibit that the function of one gut microbiome in epilepsy is diverse in different subtypes of epilepsy. As previous observational studies did not focus on the specific subtype of epilepsy, the association identified in our MR analysis may be more specific and robust. Moreover, although confounders such as antibiotic use have been controlled in most studies, previous observational studies of Proteobacteria and epilepsy may be affected by other influencing factors such as diet or age (Sullivan et al., 2001; Greenhalgh et al., 2016; Rinninella et al., 2019).

Family Veillonellaceae, which belongs to the Firmicutes phylum, is a potential risk factor for childhood absence epilepsy. Similar to previous results, the abundance of Firmicutes is increased in patients with DRE (Xie et al., 2017; Peng et al., 2018; Lee et al., 2021). Furthermore, a relatively increased level of Firmicutes in other neurological disorders, such as Parkinson’s disease, has been reported (Bedarf et al., 2017). Although direct evidence between Veillonellaceae and epilepsy is lacking, the family Veillonellaceae is positively related to non-social fear behavior in infants, which requires further elucidation based on a subsequent longitudinal study of endocrine, metabolites, and immune alteration (Carlson et al., 2021). Family Veillonellaceae is also negatively correlated with orientation and delayed recall scores in anamnestic mild cognitive impairment research (Liu et al., 2021). Moreover, Veillonellaceae can influence normal brain functions and is involved in several neurological disorders. From another perspective, *Ruminococcus*, another microbiome belonging to the Firmicutes phylum, reportedly has a lower level of N-acetyl aspartic acid (NAA) and serotonin (Bedarf et al., 2017; Mudd et al., 2017). However, NAA is important for neuronal health. As serotonin can inhibit the T-type calcium channel with reduced bursting electrical activity, increased *Ruminococcus* may sensitize epileptiform discharge because of decreased NAA and serotonin (Petersen et al., 2017). Whether Veillonellaceae influences childhood absence epilepsy by modulating neurotransmitter levels, similarly with *Ruminococcus*, is also worth exploring.

Additionally, our MR analysis identified the class Betaproteobacteria and order Burkholderia, which belong to the Proteobacteria phylum, are potential protective factors of juvenile myoclonic epilepsy. Previous associations between the microbiome and the phylum Proteobacteria have been widely reported, as discussed above and summarized in Supplementary Table 9. Alterations in the Proteobacteria phylum in patients with epilepsy are not consistent. As a common phylum in the human body, Proteobacteria are related to diseases such as obesity (Shin et al., 2015; Greenhalgh et al., 2016). Although *Escherichia* particularly *E. coli*, *Salmonella*, and *Vibrio*, which belong to the Gammaproteobacteria class, are notorious pathogens, the Proteobacteria phylum should not be considered a risk factor for all neurological diseases (Singh et al., 2015). A more specific association at the class, order, or family level of the phylum Proteobacteria is absent. Unfortunately, as for mechanisms underlying this association, studies that have identified a direct correlation among juvenile myoclonic epilepsy and class Betaproteobacteria and order Burkholderial are lacking. From the MGB axis perspective, the gut microbiota is associated with neurodevelopment, and a recent observational study has reported that gut microbiota such as Bifidobacterium, Bacteroidetes, and Lachnospiraceae can influence neurodevelopment in infants (Oliphant et al., 2021; Beghetti et al., 2022). The neurodevelopmental modulation capability of the gut microbiota may be correlated with the release of neuroactive substances, short-chain fatty acids (SCFAs), and alterations in intestinal or blood-brain barrier integrity alteration (Louis et al., 2010; Hsiao et al., 2013; Sarkar et al., 2016). As the association between juvenile myoclonic epilepsy and neurodevelopment has been discussed previously, the causality of class Betaproteobacteria or order Burkholderial and juvenile myoclonic epilepsy may be relevant to their neurodevelopment modulation capability (Lin et al., 2014). Additionally, the effect of each gut microbiome on epilepsy may not rely on one specific pathway but an integrative model including the immune system, nervous system, neurotransmitters, SCFAs, and the hypothalamic–pituitary–adrenal axis (Ding et al., 2021). Another interesting result of class Melainabacteria in generalized epilepsy with tonic-clonic seizures is that the phylum Melainabacteria has been identified as an accurate biomarker of zinc status (Chen et al., 2021). Because zinc levels are associated with neurodevelopment, Melainabacteria may influence generalized epilepsy by modulating zinc levels. Additionally, Melainabacteria, which interacts with other gut microbiomes and exerts an influence on the human body, is also essential for gut biodiversity. Lastly, after our MR analysis of the gut microbiota, the causality of gut microbiota and the three subtypes of generalized epilepsy, childhood absence epilepsy, juvenile myoclonic epilepsy, and generalized epilepsy with tonic-clonic seizures, are identified without any focal epilepsy. A possible reason for this result is that the gut microbiota releases factors to the systemic circulation with a potential role in triggering an immune response or impacting the permeability of the blood-brain barrier, functioning in the bilateral brain rather than a focal area (Logsdon et al., 2018).

Regarding gut metabolites, TMAO may be generated from L-carnitine, betaine, choline, and other choline-containing compounds that participate in the gut microbiome and hepatic flavin-dependent monooxygenases (Zeisel and Warrier, 2017). The relationship between TMAO and its predecessors with multiple chronic diseases, such as cardiovascular diseases and cancer, has also been previously studied (Cho and Caudill, 2017). The neuroprotective function of dietary choline and the therapeutic potential of carnitine in multiple neurological disorders, including epilepsy, have been reviewed previously (Blusztajn et al., 2017; Maldonado et al., 2020). Moreover, in a kainite-induced temporal lobe epilepsy model, acetyl-L-carnitine exerts its anticonvulsant effect by ameliorating oxidative stress, pyroptosis, and neuroinflammation (Tashakori-Miyanroudi et al., 2022). However, after multiple testing corrections of our MR analysis, the causality of genetically predicted TMAO, choline, carnitine, and betaine on epilepsy is not determined. A previous MR analysis has also revealed no direct causality of TMAO in Alzheimer’s disease (Zhuang et al., 2021). Hence, the previous association between TMAO and multiple neurological disorders may be interfered with by confounders. The role of TMAO in the CNS of the human body requires further investigation.

This MR study has multiple advantages over previous studies. First, this is the first MR to explore the potential causal relationship between the gut microbiota, metabolites, and epilepsy. Second, our MR study is based on the largest GWAS-summary-level data on the gut microbiome from the MiBioGen study. A bidirectional MR analysis is conducted to ensure the robustness of our results. Several microbiomes have been identified as potential therapeutic targets in patients with epilepsy. As the gut microbiota GWAS dataset is based on three ancestries, the generalizability of our results applies to different populations. The non-significant causalities of our results also provide important information that previous observational studies may had been easily interfered with by confounders. Lastly, whether gut metabolites such as TMAO are potential predisposing factors for epilepsy has also been studied. Our MR analysis indicates that specific gut microbiome functions should be considered in a specific subtype of epilepsy, rather than from a general perspective.

Nevertheless, this study has some limitations. First, as SNPs in the MiBioGen study less than 5 × 10^–8^ are not sufficient for MR analysis, the significance level of gut microbiota IV selection is set at 1 × 10^–5^ instead of 5 × 10^–8^, which is the same as that in a previous publication (Ni et al., 2021). However, the F-statistics are guaranteed to be > 10 to exclude potential weak instrument bias and make the statistical results more robust (Burgess and Thompson, 2011). Second, as the epilepsy ILAE GWAS dataset lacks exposure levels and periods, the effect of gut microbiomes and metabolites on epilepsy exposed to different levels or timings must be further ascertained. Third, although previous observational studies mainly focused on the relationship between the gut microbiota and DRE, MR has not been conducted to explore the causality between gut microbiota, metabolites, and DRE due to the lack of GWAS summary-level data on DRE, which is worth researching in future. Fourth, direct mechanistic research to support our findings is still lacking. To obtain more direct evidence of the relationship between the gut microbiota and epilepsy, more research is required to explore the effects of these bacteria on the immune response, blood-brain barrier permeability, neuronal excitability, and brain development. Lastly, the gut microbiota is easily influenced by environmental factors, such as diet. However, horizontal pleiotropy is excluded from our MR analysis based on the following two sensitivity analyses: MR-Egger and MR-PRESSO results.

Collectively, we comprehensively analyzed the potential causal relationship among the gut microbiota, metabolites, and epilepsy. This bi-directional MR study ascertained the predisposing or protective effects of the family Veillonellaceae on childhood absence epilepsy, class Melainabacteria on generalized epilepsy with tonic-clonic seizures, class Betaproteobacteria, and order Burkholderials on juvenile myoclonic epilepsy, which are promising gut biomarkers and novel therapeutic targets of epilepsy.

## Data availability statement

The original contributions presented in this study are included in the article/Supplementary material, further inquiries can be directed to the corresponding author/s.

## Author contributions

YO, YC, and LL designed the study. YO and YC performed the analysis and drafted the manuscript. GW and YS participated in revising the manuscript. LL, ZY, and BX reviewed the manuscript for its intellectual content and revise the entire work. All authors read and approved the final manuscript.

## Footnotes

1
https://mrcieu.github.io/TwoSampleMR

2
https://gwas.mrcieu.ac.uk/

## Supplementary material

Supplementary Figure 1 Mendelian randomization leave-one-out sensitivity analysis of significant results. **(A)** Leave-one-out analysis of the family Veillonellaceae on childhood absence epilepsy. **(B)** Leave-one-out analysis of the class Melainabacteria on generalized epilepsy with tonic-clonic seizures. Leave-one-out analysis of **(C)** the class Betaproteobacteria and **(D)** the order Burkholderiales on juvenile myoclonic epilepsy.

Supplementary Figure 2 Funnel plots demonstrating significant results. **(A)** Funnel plots of the family Veillonellaceae on childhood absence epilepsy. **(B)** Funnel plots of the class Melainabacteria on generalized epilepsy with tonic-clonic seizures. Funnel plots of **(C)** the class Betaproteobacteria and **(D)** the order Burkholderiales on juvenile myoclonic epilepsy.

## Abbreviations

CNS: central nervous system
DRE: drug-resistant epilepsy
FMT: fecal microbiota transplantation
GWAS: genome-wide association study
ILAE: International League Against Epilepsy
IVs: instrumental variables
IVW: inverse variance weighted median
KD: ketogenic diet
MGB: microbiota–gut–brain
MR: Mendelian randomization
NAA: N-acetyl aspartic acid
RCT: randomized controlled trial
SNP: single nucleotide polymorphism
TMAO: trimethylamine N-oxide.
