# Supplementary material for: Genetically proxied gut microbiota, gut metabolites with risk of epilepsy and the subtypes: A bi-directional Mendelian randomization study
Source: Front Mol Neurosci. 2022 Nov 3;15:994270. doi: 10.3389/fnmol.2022.994270 (PMC9669914; doi:10.3389/fnmol.2022.994270)
Supplement: Supplementary file 1 [file Table_1.docx]

**Supplement Table 1. IVs used in this MR**

| **Exposeure** | **Outcome**  **(ID)** | **SNP** | **Chr** | **Position** | **Effect**  **allele** | **Other**  **allele** | **Exposure** | | | | **Outcome** | | | |
| --- | --- | --- | --- | --- | --- | --- | --- | --- | --- | --- | --- | --- | --- | --- |
|  |  |  |  |  |  |  | **Beta** | **SE** | **P-value** | **Sample**  **size** | **Beta** | **SE** | **P-value** | **Sample**  **size** |
| Family  Veillonellaceae | ieu-b-13 | rs11700976 | 21 | 35576123 | C | A | 0.05033 | 0.01138 | 9.25 x10^-06^ | 15971 | 0.00081 | 0.00156 | 6.00 x10^-01^ | 30470 |
|  |  | rs12741784 | 1 | 50088819 | C | T | -0.06215 | 0.01193 | 1.28 x10^-07^ | 15867 | -0.00095 | 0.00159 | 5.50 x10^-01^ | 30470 |
|  |  | rs1442060 | 4 | 46366067 | G | A | -0.05140 | 0.01121 | 4.51 x10^-06^ | 15976 | -0.00268 | 0.00157 | 8.70 x10^-02^ | 30470 |
|  |  | rs2175069 | 4 | 23317124 | A | G | -0.05268 | 0.01149 | 4.64 x10^-06^ | 15976 | -0.00145 | 0.00159 | 3.60 x10^-01^ | 30470 |
|  |  | rs2561116 | 5 | 38348199 | T | G | -0.08362 | 0.01872 | 7.89 x10^-06^ | 15976 | -0.00167 | 0.00260 | 5.20 x10^-01^ | 30470 |
|  |  | rs2585520 | 13 | 78785141 | G | T | -0.09050 | 0.02004 | 5.27 x10^-06^ | 15501 | -0.00665 | 0.00274 | 1.50 x10^-02^ | 30470 |
|  |  | rs4263802 | 8 | 138319508 | G | A | 0.05094 | 0.01149 | 7.45 x10^-06^ | 15976 | 0.00270 | 0.00157 | 8.60 x10^-02^ | 30470 |
|  |  | rs4461038 | 15 | 78546996 | A | G | -0.05550 | 0.01195 | 3.73 x10^-06^ | 15973 | -0.00164 | 0.00160 | 3.10 x10^-01^ | 30470 |
|  |  | rs4797169 | 18 | 462180 | T | C | 0.05878 | 0.01283 | 4.49 x10^-06^ | 15974 | 0.00036 | 0.00179 | 8.40 x10^-01^ | 30470 |
|  |  | rs6909981 | 6 | 75534152 | C | T | -0.06387 | 0.01418 | 5.48 x10^-06^ | 15976 | -0.00212 | 0.00192 | 2.70 x10^-01^ | 30470 |
|  |  | rs9345168 | 6 | 92380922 | C | A | 0.05091 | 0.01133 | 8.49 x10^-06^ | 15976 | 0.00169 | 0.00154 | 2.70 x10^-01^ | 30470 |
| Class  Melainabacteria | ieu-b-16 | rs10738747 | 9 | 26184578 | G | A | 0.08146 | 0.01844 | 9.96 x10^-06^ | 5884 | -0.00141 | 0.00087 | 1.10 x10^-01^ | 29905 |
|  |  | rs11150282 | 16 | 80493705 | T | C | 0.09895 | 0.01970 | 6.03 x10^-07^ | 5873 | -0.00248 | 0.00093 | 7.60 x10^-03^ | 29905 |
|  |  | rs113884518 | 9 | 24648997 | T | C | -0.20535 | 0.04550 | 8.06 x10^-06^ | 4442 | 0.00104 | 0.00197 | 6.00 x10^-01^ | 29905 |
|  |  | rs367480 | 11 | 2937631 | G | A | -0.08376 | 0.01858 | 8.20 x10^-06^ | 5887 | 0.00031 | 0.00088 | 7.20 x10-^01^ | 29905 |
|  |  | rs4129395 | 9 | 115975389 | G | A | 0.08962 | 0.01851 | 1.48 x10^-06^ | 5887 | -0.00069 | 0.00088 | 4.30 x10^-01^ | 29905 |
|  |  | rs789069 | 18 | 1008278 | A | C | -0.10354 | 0.02343 | 6.85 x10^-06^ | 5879 | 0.00175 | 0.00114 | 1.20 x10^-01^ | 29905 |
|  |  | rs9864379 | 3 | 14306949 | T | C | -0.15973 | 0.02926 | 5.36 x10^-08^ | 5777 | 0.00302 | 0.00140 | 3.10 x10^-02^ | 29905 |
| Class  Betaproteobacteria | ieu-b-17 | rs11128180 | 3 | 70592215 | A | G | 0.05929 | 0.01288 | 3.67 x10^-06^ | 17151 | -0.00521 | 0.00220 | 1.80 x10^-02^ | 30858 |
|  |  | rs1511453 | 4 | 23588462 | A | G | 0.09235 | 0.01992 | 4.76 x10^-06^ | 17085 | -0.00409 | 0.00382 | 2.80 x10^-01^ | 30858 |
|  |  | rs1928341 | 1 | 153240013 | A | G | 0.05256 | 0.01104 | 2.02 x10^-06^ | 17150 | -0.00251 | 0.00191 | 1.90 x10^-01^ | 30858 |
|  |  | rs2321387 | 13 | 58689340 | G | A | -0.04946 | 0.01094 | 5.80 x10^-06^ | 17149 | 0.00354 | 0.00186 | 5.70 x10^-02^ | 30858 |
|  |  | rs2613606 | 7 | 111285025 | C | T | -0.05131 | 0.01091 | 2.20 x10^-06^ | 17146 | 0.00228 | 0.00187 | 2.20 x10^-01^ | 30858 |
|  |  | rs320161 | 9 | 104899117 | A | G | 0.05723 | 0.01262 | 7.33 x10^-06^ | 17035 | -0.00389 | 0.00213 | 6.80 x10^-02^ | 30858 |
|  |  | rs4033856 | 4 | 45642485 | C | T | 0.08324 | 0.01672 | 5.17 x10^-07^ | 15945 | -0.00394 | 0.00293 | 1.80 x10^-01^ | 30858 |
|  |  | rs56386628 | 8 | 112732674 | C | T | -0.06223 | 0.01357 | 5.87 x10^-06^ | 17147 | 0.00226 | 0.00231 | 3.30 x10^-01^ | 30858 |
|  |  | rs6087811 | 20 | 30596130 | T | G | -0.09782 | 0.01985 | 7.44 x10^-07^ | 16184 | 0.00055 | 0.00352 | 8.80 x10^-01^ | 30858 |
|  |  | rs62395635 | 5 | 173497796 | T | C | 0.10974 | 0.02360 | 2.94 x10^-06^ | 15108 | 0.00082 | 0.00390 | 8.30 x10^-01^ | 30858 |
|  |  | rs9964679 | 18 | 26569901 | A | G | 0.05291 | 0.01149 | 4.85 x10^-06^ | 17151 | -0.00178 | 0.00201 | 3.80 x10^-01^ | 30858 |
| Order  Burkholderiales | ieu-b-17 | rs1511453 | 4 | 23588462 | A | G | 0.09110 | 0.01995 | 8.00 x10^-06^ | 17032 | -0.00409 | 0.00382 | 2.80 x10^-01^ | 30858 |
|  |  | rs1928341 | 1 | 153240013 | A | G | 0.05078 | 0.01106 | 4.52 x10^-06^ | 17096 | -0.00251 | 0.00191 | 1.90 x10^-01^ | 30858 |
|  |  | rs2321387 | 13 | 58689340 | G | A | -0.05085 | 0.01096 | 3.26 x10^-06^ | 17095 | 0.00354 | 0.00186 | 5.70 x10^-02^ | 30858 |
|  |  | rs2613606 | 7 | 111285025 | C | T | -0.04996 | 0.01093 | 4.13 x10^-06^ | 17092 | 0.00228 | 0.00187 | 2.20 x10^-01^ | 30858 |
|  |  | rs4033856 | 4 | 45642485 | C | T | 0.08334 | 0.01675 | 5.67 x10^-07^ | 15897 | -0.00394 | 0.00293 | 1.80 x10^-01^ | 30858 |
|  |  | rs6087811 | 20 | 30596130 | T | G | -0.10159 | 0.01988 | 2.88 x10^-07^ | 16135 | 0.00055 | 0.00352 | 8.80 x10^-01^ | 30858 |
|  |  | rs62395635 | 5 | 173497796 | T | C | 0.10991 | 0.02364 | 2.90 x10^-06^ | 15062 | 0.00082 | 0.00390 | 8.30 x10^-01^ | 30858 |
|  |  | rs7638039 | 3 | 70588939 | T | C | 0.05810 | 0.01267 | 4.84 x10^-06^ | 17078 | -0.00499 | 0.00218 | 2.20 x10^-02^ | 30858 |
|  |  | rs9964679 | 18 | 26569901 | A | G | 0.05251 | 0.01150 | 6.06 x10^-06^ | 17097 | -0.00178 | 0.00201 | 3.80 x10^-01^ | 30858 |
