# Supplementary material for: Genetically proxied gut microbiota, gut metabolites with risk of epilepsy and the subtypes: A bi-directional Mendelian randomization study
Source: Front Mol Neurosci. 2022 Nov 3;15:994270. doi: 10.3389/fnmol.2022.994270 (PMC9669914; doi:10.3389/fnmol.2022.994270)
Supplement: Supplementary file 2 [file Table_2.docx]

**Supplement Table 2. p-value of MR steiger test, heterogeneity test, pleiotropy test and F statistic**

| **Exposure** | **Outcome**  **(ID)** | **Heterogeneity test** | | **Pleiotropy test** | | **MR steiger test** | **F** |
| --- | --- | --- | --- | --- | --- | --- | --- |
|  |  | **IVW test** | **MR-Egger regression** | **MR-PRESSO global test** | **MR pleiotropy test** |  |  |
| family.Veillonellaceae | ieu-b-13 | 0.9290 | 0.8972 | 0.9440 | 0.6948 | 9.6782 x10^-26^ | 20.73 |
| class.Melainabacteria | ieu-b-16 | 0.6634 | 0.5365 | 0.3670 | 0.9336 | 3.040 x10^-30^ | 18.04 |
| class.Betaproteobacteria | ieu-b-17 | 0.8393 | 0.9548 | 0.8750 | 0.1495 | 4.5298 x10^-23^ | 20.10 |
| order.Burkholderiales | ieu-b-17 | 0.7468 | 0.9052 | 0.7750 | 0.1709 | 1.2296 x10^-19^ | 20.29 |
