# Supplementary material for: Genetically proxied gut microbiota, gut metabolites with risk of epilepsy and the subtypes: A bi-directional Mendelian randomization study
Source: Front Mol Neurosci. 2022 Nov 3;15:994270. doi: 10.3389/fnmol.2022.994270 (PMC9669914; doi:10.3389/fnmol.2022.994270)
Supplement: Supplementary file 3 [file Table_9.docx]

| Phylum | Class | Family | Genus |
| --- | --- | --- | --- |
| Proteobacteria↑  Proteobacteria-KD↓ | \ | \ | Sutterella↑  Escherichia coli-KD↑  Cronobacter-KD↓ |
| Fusobacterial↑ | \ | \ | Fusobacterium↑ |
| Bacteroidetes↑  Bacteroidetes-KD↑  Bacteroidetes-DRE↓ | \ | \ | Alloprevotella↑  Bacteroides↑  Alloprevotella-KD↑  Bacteroides-KD↑  Prevotella-KD↑  Alistipes-KD↓  Barnesiella-KD↓  Bacteroides-DRE↓↑ |
| Firmicute-DRE↑  Firmicute-KD↓ | Negativicutes-DRE↑ | Lachnospiraceae-KD↓  Enterococcaceae-KD↓ | Megasphaera↑  Ruminococcus↑  Ruminococcus-DRE↑  Enterococcus↑  Subdoligranulum-KD↑  Dialister-KD↑  Erysipelatoclostridium-KD↓  Streptococcus-KD↓  Enterococcus-KD↓  Ruminiclostridium-KD↓  Enterococcus-DRE↓  Anaerostipes-DRE↓  Blautia-DRE↓ |
| Verrucomicrobia-DRE↑ | \ | \ | Akkermansia-KD↓ |
| Actinobacteria-DRE↓↑ | \ | Bifidobacterium -KD↑  Actinomyces-KD↓  Bifidobacterium-KD↓ | Actinomyces↑  Bifidobacteroides-DRE↓  Bifidobacterium-KD↓↑ |

Supplement table 9: Summarization of gut microbiota changes in previous observational studies of epilepsy patients

Legend: “↑”：gut microbiome increases; “↓” gut microbiome decreases; “↓↑” gut microbiome increases or decreases reported in different publications; xxx-DRE: gut microbiome alteration in drug refractory epilepsy patients; xxx-KD: gut microbiome change after ketogenic diet
