# Supplementary material for: Genetically proxied gut microbiota, gut metabolites with risk of epilepsy and the subtypes: A bi-directional Mendelian randomization study
Source: Front Mol Neurosci. 2022 Nov 3;15:994270. doi: 10.3389/fnmol.2022.994270 (PMC9669914; doi:10.3389/fnmol.2022.994270)
Supplement: Supplementary file 4 [file Table_10.docx]

**Supplement table 10: STROBE-MR checklist of recommended items to address in reports of Mendelian randomization studies**^1^ ^2^

| **Item No.** | **Section** | **Checklist item** | **Page No.** | **Relevant text from manuscript** |
| --- | --- | --- | --- | --- |
| 1 | **TITLE and ABSTRACT** | Indicate Mendelian randomization (MR) as the study’s design in the title and/or the abstract if that is a main purpose of the study | 1,2 | Title and abstract |
|  | **INTRODUCTION** |  | 2,3 | Page 2 Line 121-Page 3 Line 214 |
| 2 | **Background** | Explain the scientific background and rationale for the reported study. What is the exposure? Is a potential causal relationship between exposure and outcome plausible? Justify why MR is a helpful method to address the study question | 2 | Page 2 Line 121-Page 5 Line 203 |
| 3 | **Objectives** | State specific objectives clearly, including pre-specified causal hypotheses (if any). State that MR is a method that, under specific assumptions, intends to estimate causal effects | 2,3 | Page 2 Line 204-Page 3 Line 214 |
|  | **METHODS** |  |  |  |
| 4 | **Study design and data sources** | Present key elements of the study design early in the article. Consider including a table listing sources of data for all phases of the study. For each data source contributing to the analysis, describe the following: |  |  |
|  | a) | Setting: Describe the study design and the underlying population, if possible. Describe the setting, locations, and relevant dates, including periods of recruitment, exposure, follow-up, and data collection, when available. | 3,4 | Page 3 Line 219-Page 4 Line 335; Figure 1A |
|  | b) | Participants: Give the eligibility criteria, and the sources and methods of selection of participants. Report the sample size, and whether any power or sample size calculations were carried out prior to the main analysis | / | Table 1; Original GWAS studies of ILAE, MiBioGen, and gut metabolites. |
|  | c) | Describe measurement, quality control and selection of genetic variants | 4 | Page 4 Line 338-Page 4 Line 385 |
|  | d) | For each exposure, outcome, and other relevant variables, describe methods of assessment and diagnostic criteria for diseases | / | Original GWAS studies of ILAE, MiBioGen, and gut metabolites |
|  | e) | Provide details of ethics committee approval and participant informed consent, if relevant | / | Not applicable |
| 5 | **Assumptions** | Explicitly state the three core IV assumptions for the main analysis (relevance, independence and exclusion restriction) as well assumptions for any additional or sensitivity analysis | 4 | Page 4 Line 390-Page 4 Line 396; Figure 1B |
| 6 | **Statistical methods: main analysis** | Describe statistical methods and statistics used |  |  |
|  | a) | Describe how quantitative variables were handled in the analyses (i.e., scale, units, model) | / | Not applicable |
|  | b) | Describe how genetic variants were handled in the analyses and, if applicable, how their weights were selected | 4,5 | Page 4 Line 397-Page 5 Line 435 |
|  | c) | Describe the MR estimator (e.g. two-stage least squares, Wald ratio) and related statistics. Detail the included covariates and, in case of two-sample MR, whether the same covariate set was used for adjustment in the two samples | 4 | Page 4 Line 397-Page 5 Line 425 |
|  | d) | Explain how missing data were addressed | / | Not applicable |
|  | e) | If applicable, indicate how multiple testing was addressed | 4,5 | Page 4 Line 397-Page 5 Line 435 |
| 7 | **Assessment of assumptions** | Describe any methods or prior knowledge used to assess the assumptions or justify their validity | 5 | Page 5 Line 442-Page 5 Line 450; Figure 1; Table 2 |
| 8 | **Sensitivity analyses and additional analyses** | Describe any sensitivity analyses or additional analyses performed (e.g. comparison of effect estimates from different approaches, independent replication, bias analytic techniques, validation of instruments, simulations) | 5 | Page 5 Line 442-Page 5 Line 450 |
| 9 | **Software and pre-registration** |  |  |  |
|  | a) | Name statistical software and package(s), including version and settings used | 3 | Page 3 Line 225-Page 3 Line 266 |
|  | b) | State whether the study protocol and details were pre-registered (as well as when and where) | / | Not applicable |
|  | **RESULTS** |  |  |  |
| 10 | **Descriptive data** |  |  |  |
|  | a) | Report the numbers of individuals at each stage of included studies and reasons for exclusion. Consider use of a flow diagram | / | Not applicable |
|  | b) | Report summary statistics for phenotypic exposure(s), outcome(s), and other relevant variables (e.g. means, SDs, proportions) | / | Table 1 |
|  | c) | If the data sources include meta-analyses of previous studies, provide the assessments of heterogeneity across these studies | / | Not applicable |
|  | d) | For two-sample MR:  i.  Provide justification of the similarity of the genetic variant-exposure associations between the exposure and outcome samples  ii.  Provide information on the number of individuals who overlap between the exposure and outcome studies | / | Table 1 |
| 11 | **Main results** |  |  |  |
|  | a) | Report the associations between genetic variant and exposure, and between genetic variant and outcome, preferably on an interpretable scale | 5,6 | Page 5 Line 466-Page 6 Line 627; Table 2 |
|  | b) | Report MR estimates of the relationship between exposure and outcome, and the measures of uncertainty from the MR analysis, on an interpretable scale, such as odds ratio or relative risk per SD difference | 5 | Page 5 Line 483-Page 5 Line 498; Table 2 |
|  | c) | If relevant, consider translating estimates of relative risk into absolute risk for a meaningful time period | / | Not applicable |
|  | d) | Consider plots to visualize results (e.g. forest plot, scatterplot of associations between genetic variants and outcome versus between genetic variants and exposure) | / | Figure 2; Figure 3 |
| 12 | **Assessment of assumptions** |  |  |  |
|  | a) | Report the assessment of the validity of the assumptions | 5 | Page 5 Line 499-Page 5 Line 509; Table 2 |
|  | b) | Report any additional statistics (e.g., assessments of heterogeneity across genetic variants, such as *I^2^*, Q statistic or E-value) | 5 | Page 5 Line 499-Page 5 Line 509; Table 2; Supplement Figure 1; Supplement Figure 2 |
| 13 | **Sensitivity analyses and additional analyses** |  |  |  |
|  | a) | Report any sensitivity analyses to assess the robustness of the main results to violations of the assumptions | 5 | Page 5 Line 499-Page 5 Line 509; Table 2; |
|  | b) | Report results from other sensitivity analyses or additional analyses | 5 | Page 5 Line 499-Page 5 Line 509; Table 2; Supplement Figure 1; Supplement Figure 2 |
|  | c) | Report any assessment of direction of causal relationship (e.g., bidirectional MR) | 5 | Page 5 Line 512-Page 5 Line 521 |
|  | d) | When relevant, report and compare with estimates from non-MR analyses | / | Not applicable |
|  | e) | Consider additional plots to visualize results (e.g., leave-one-out analyses) | / | Supplement Figure 1; Supplement Figure 2 |
|  | **DISCUSSION** |  |  |  |
| 14 | **Key results** | Summarize key results with reference to study objectives | 9 | Page 9 Line 896-Page 9 Line 903 |
| 15 | **Limitations** | Discuss limitations of the study, taking into account the validity of the IV assumptions, other sources of potential bias, and imprecision. Discuss both direction and magnitude of any potential bias and any efforts to address them | 9 | Page 9 Line 870-Page 9 Line 895 |
| 16 | **Interpretation** |  |  |  |
|  | a) | Meaning: Give a cautious overall interpretation of results in the context of their limitations and in comparison with other studies | 6 | Page 6 Line 632-Page 6 Line 670 |
|  | b) | Mechanism: Discuss underlying biological mechanisms that could drive a potential causal relationship between the investigated exposure and the outcome, and whether the gene-environment equivalence assumption is reasonable. Use causal language carefully, clarifying that IV estimates may provide causal effects only under certain assumptions | 8,9 | Page 8 Line 743-Page 9 Line 850 |
|  | c) | Clinical relevance: Discuss whether the results have clinical or public policy relevance, and to what extent they inform effect sizes of possible interventions | 9 | Page 9 Line 896-Page 9 Line 903 |
| 17 | **Generalizability** | Discuss the generalizability of the study results (a) to other populations, (b) across other exposure periods/timings, and (c) across other levels of exposure | 9 | Page 9 Line 851-Page 9 Line 869 |
|  | **OTHER INFORMATION** |  |  |  |
| 18 | **Funding** | Describe sources of funding and the role of funders in the present study and, if applicable, sources of funding for the databases and original study or studies on which the present study is based | 9 | Page 9 Line 921-Page 9 Line 931 |
| 19 | **Data and data sharing** | Provide the data used to perform all analyses or report where and how the data can be accessed, and reference these sources in the article. Provide the statistical code needed to reproduce the results in the article, or report whether the code is publicly accessible and if so, where | 9 | Page 9 Line 906-910 |
| 20 | **Conflicts of Interest** | All authors should declare all potential conflicts of interest | 9 | Page 9 Line 941-946 |

This checklist is copyrighted by the Equator Network under the Creative Commons Attribution 3.0 Unported (CC BY 3.0) license.

1. Skrivankova VW, Richmond RC, Woolf BAR, Yarmolinsky J, Davies NM, Swanson SA, et al. Strengthening the Reporting of Observational Studies in Epidemiology using Mendelian Randomization (STROBE-MR) Statement. JAMA. 2021;under review.

2. Skrivankova VW, Richmond RC, Woolf BAR, Davies NM, Swanson SA, VanderWeele TJ, et al. Strengthening the Reporting of Observational Studies in Epidemiology using Mendelian Randomisation (STROBE-MR): Explanation and Elaboration. BMJ. 2021;375:n2233.
