# Supplementary figures and images for: Genetically proxied gut microbiota, gut metabolites with risk of epilepsy and the subtypes: A bi-directional Mendelian randomization study
Source: Front Mol Neurosci. 2022 Nov 3;15:994270. doi: 10.3389/fnmol.2022.994270 (PMC9669914; doi:10.3389/fnmol.2022.994270)

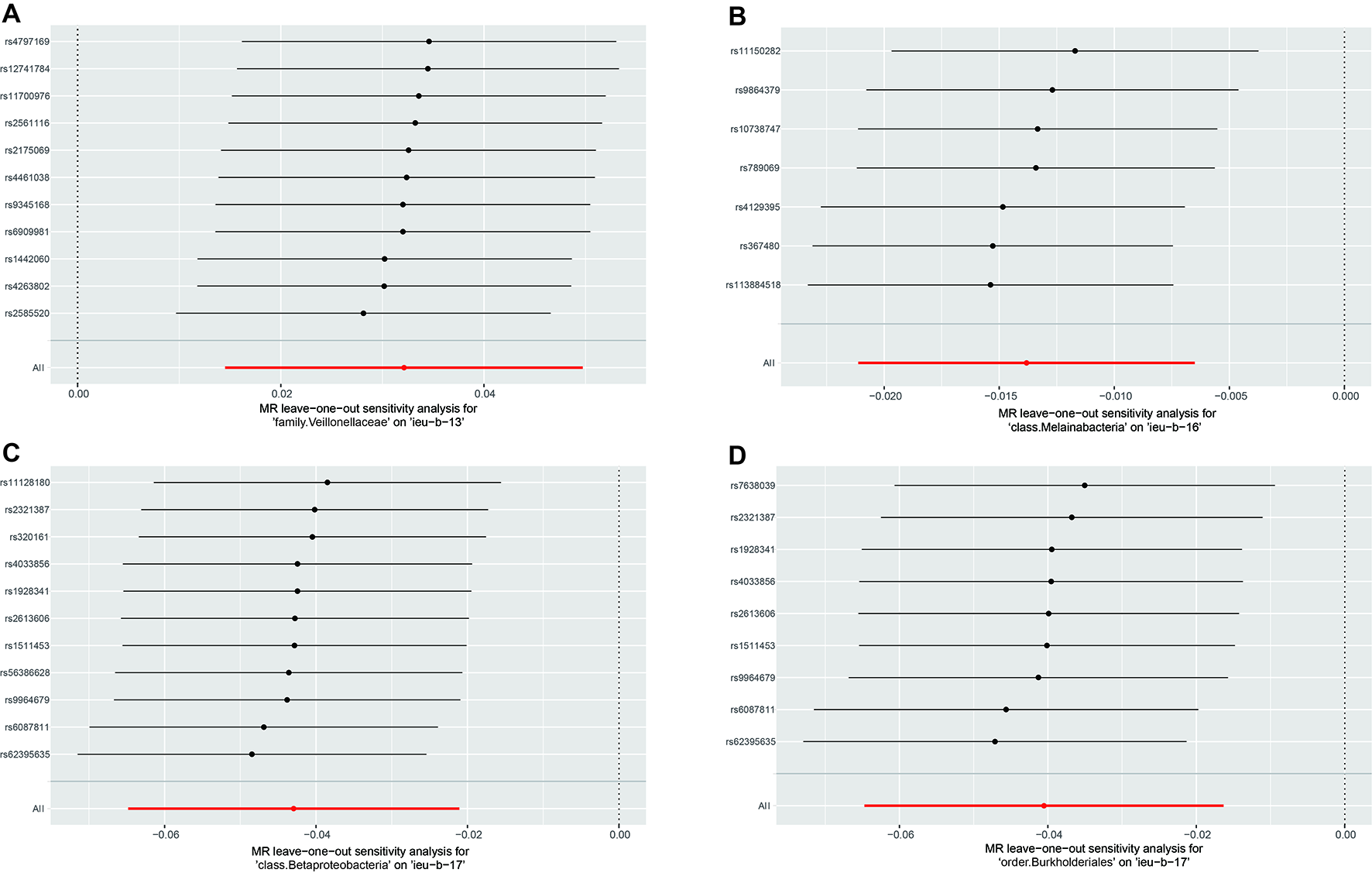

Supplement: Supplementary Figure 1 — Mendelian randomization leave-one-out sensitivity analysis of significant results. (A) Leave-one-out analysis of the family Veillonellaceae on childhood absence epilepsy. (B) Leave-one-out analysis of the class Melainabacteria on generalized epilepsy with tonic-clonic seizures. Leave-one-out analysis of (C) the class Betaproteobacteria and (D) the order Burkholderiales on juvenile myoclonic epilepsy. [file Image_1.TIF]

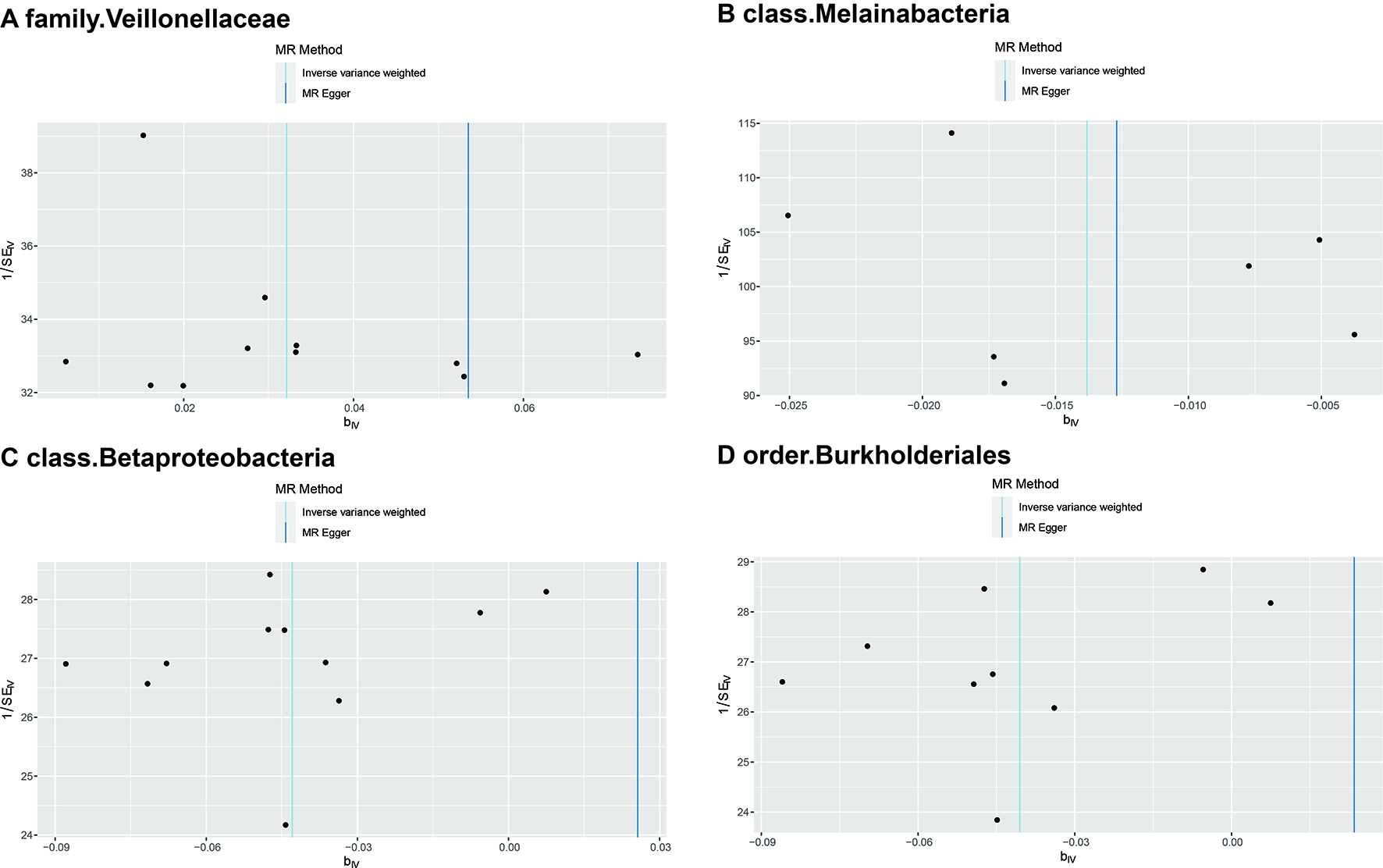

Supplement: Supplementary Figure 2 — Funnel plots demonstrating significant results. (A) Funnel plots of the family Veillonellaceae on childhood absence epilepsy. (B) Funnel plots of the class Melainabacteria on generalized epilepsy with tonic-clonic seizures. Funnel plots of (C) the class Betaproteobacteria and (D) the order Burkholderiales on juvenile myoclonic epilepsy. [file Image_2.TIF]
